# Hybrid Mold: Comparative Study of Rapid and Hard Tooling for Injection Molding Application Using Metal Epoxy Composite (MEC)

**DOI:** 10.3390/ma14030665

**Published:** 2021-02-01

**Authors:** Radhwan Hussin, Safian Sharif, Marcin Nabiałek, Shayfull Zamree Abd Rahim, Mohd Tanwyn Mohd Khushairi, Mohd Azlan Suhaimi, Mohd Mustafa Al Bakri Abdullah, Mohd Hazwan Mohd Hanid, Jerzy J. Wysłocki, Katarzyna Błoch

**Affiliations:** 1 School of Mechanical Engineering, Faculty of Mechanical Engineering, Universiti Teknology Malaysia, Johor Bahru 81310, Malaysia; azlansuhaimi@utm.my; 2 Center of Excellence Geopolymer and Green Technology (CEGeoGTech), Universiti Malaysia Perlis, Perlis 01000, Malaysia; mustafa_albakri@unimap.edu.my (M.M.A.B.A.); hazwanhanid@unimap.edu.my (M.H.M.H.); 3 Faculty of Mechanical Engineering Technology, Universiti Malaysia Perlis, Perlis 01000, Malaysia; 4 Department of Physics, Częstochowa University of Technology, 42-200 Częstochowa, Poland; wyslocki.jerzy@wip.pcz.pl (J.J.W.); katarzyna.bloch@wip.pcz.pl (K.B.); 5 IMU Centre for Life-Long Learning (ICL), International Medical University, Kuala Lumpur 57000, Malaysia; tanwynk@gmail.com; 6 Faculty of Chemical Engineering Technology, Universiti Malaysia Perlis, Perlis 01000, Malaysia

**Keywords:** hybrid mold, rapid tooling (RT), metal epoxy composite (MEC), material properties, injection molding process

## Abstract

The mold-making industry is currently facing several challenges, including new competitors in the market as well as the increasing demand for a low volume of precision moldings. The purpose of this research is to appraise a new formulation of Metal Epoxy Composite (MEC) materials as a mold insert. The fabrication of mold inserts using MEC provided commercial opportunities and an alternative rapid tooling method for injection molding application. It is hypothesized that the addition of filler particles such as brass and copper powders would be able to further increase mold performance such as compression strength and thermal properties, which are essential in the production of plastic parts for the new product development. This study involved four phases, which are epoxy matrix design, material properties characterization, mold design, and finally the fabrication of the mold insert. Epoxy resins filled with brass (EB) and copper (EC) powders were mixed separately into 10 wt% until 30 wt% of the mass composition ratio. Control factors such as degassing time, curing temperature, and mixing time to increase physical and mechanical properties were optimized using the Response Surface Method (RSM). The study provided optimum parameters for mixing epoxy resin with fillers, where the degassing time was found to be the critical factor with 35.91%, followed by curing temperature with 3.53% and mixing time with 2.08%. The mold inserts were fabricated for EB and EC at 30 wt% based on the optimization outcome from RSM and statistical ANOVA results. It was also revealed that the EC mold insert offers better cycle time compared to EB mold insert material.

## 1. Introduction

Currently, plastic parts for various applications are produced mainly through the injection molding process under heat and pressure conditions to form the product into the desired shape and size [1,2]. In general, molds for plastic injection molding are usually made of tool steel through the machining process using a CNC (Computer Numerical Control) precision machine [3,4,5]. Toolmakers need to invest in expensive equipment, such as CNC machine tools, an Electro-Discharge Machine (EDM), drilling machine and metrology equipment, which have high flexibility for small-to-medium batch production and mold component fabrication [6]. The dimensional accuracy of the tooling and fabrication process depends on the strict requirements of the final product which requires high precision for complex geometry and fine surface finishing [7]. In addition, the market also demands products of a higher quality, cheaper costs, shorter product development cycles, and that fulfill the environmental requirements for sustainability [3,8]. Therefore, innovations in appliance methods and materials need to be adopted to meet the product manufacturing cycle requirements [9,10,11]. 

**Figure 1:**
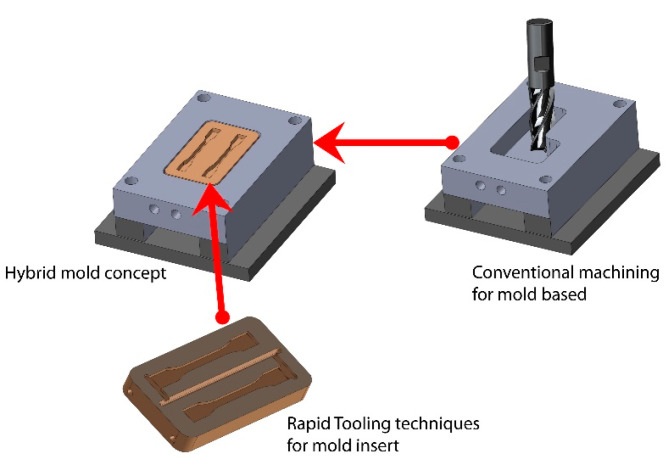
Concept of a hybrid mold.

In the last two decades, in order to meet the new trends within the plastics industry, the concept of a hybrid mold (Figure 1) has been developed for injection molding applications. A hybrid mold is a novel method in the fabrication of injection molds which combines the conventional machining for mold-based and Rapid Tooling (RT) techniques for mold inserts (core and cavity inserts) [10,12,13]. The advantages of this type of mold are efficiency in minimizing waste and energy consumption; agility to customize and ease of flexibility to change and incorporate design concepts [14,15,16]. The advantages of this type of mold are efficiency in minimizing waste and energy consumption; agility to customize and ease of flexibility to change and incorporate design concepts [2,3,8]. Manufacturers have sought the non-conventional process for tooling fabrication which includes RT and explored alternative materials with faster delivery, increased quality, reduced product development time, and compatible with global trends [10].

A common route for fabricating molding blocks and mold inserts is through the vacuum casting process of the Metal Epoxy Composite (MEC) [17,18], whereby epoxy-based material was mixed with the metal fillers (aluminum, brass, and copper) and then poured into the well-prepared pouring container. Nevertheless, the non-uniform mixing of the raw materials, the curing agent, and the presence of trapped gases may cause problems in the fabricated molding blocks and mold inserts [19,20]. To establish the best composition and mixing ratio based on percentage weight (wt%), the appropriate composition of metal fillers needs to be determined [15,21,22,23]. Pontes and Queirós [12] evaluated the performance of aluminum-filled epoxy mold inserts built by using a hybrid mold, and tested the mold insert for more than 600 shots. The material used was not just aluminum-filled epoxy, but a modified mixture of aluminum-filled epoxy with a nickel-phosphorus layer on its cavity. Tomori et al. [24] investigated the ceramic-filled epoxy tool as mold inserts for plastic injection molding. During the first 150 shots, the tool performed well without catastrophic failure, with the injection pressure and temperature being, respectively, 20 MPa (200 bar) and 220 °C. However, the results showed a significant effect in terms of the increase in surface roughness, flexural strength and thermal conductivity as a mold insert fabricated using RT methods instead of employing conventional methods. On the other hand, S. Rahmati and P. Dickens [25] pointed out that resin temperature (Tg), thermal conductivity of filler and fabrication process are important factors in considering how the molded component is affected by the molding process using mold inserts fabricated using the RT technique. There are still some difficulties in the manufacturing of plastic parts using hybrid molds which are related to the thermal properties, mechanical data and behavior of the materials which are either inaccessible or misunderstood [10]. One of the main issues encountered during the development of RT for the molding process is its low thermal conductivity, which results in slow heat transfer from the molten plastic to the coolant through the mold inserts. Rapid heating and cooling during the injection molding process can further degrade the mold inserts and consequently affect the quality of the part and dimensional accuracy [16,26].

Selection of the best composition of filler and well preparation process of mold insert will lead to enhanced mechanical properties that are capable of a higher production volume, such as hardness, strength, and cost-effective molds [15,16,27]. However, the metal fillers will perform better in the epoxy matrix with uniform dispersion as well as suspension in the mixture, and they do not sink to the bottom [15]. This is an important factor that needs to be considered in order to produce effective MEC mold inserts. In addition, there are several other factors based on the manufacturer’s guidelines and also reported by previous studies [15,23,28] such as curing temperature, curing time, composition of metal fillers based on its weight ratio, degassing time, mixing time, etc. [29], which will affect the physical, thermal and mechanical properties of the mold inserts produced. To understand the effect of these parameters, the interactions between all of these parameters to the responses should be examined. 

Response Surface Methodology (RSM) is useful in correlating the factors and responses as it is less time consuming and is able to detect the true optimum factor [30,31]. This allows a number of factors to be simultaneously evaluated and eliminates the need for a large number of independent tests that are otherwise necessary for a standard one-factor or trial and error approach [29,32].

## 2. Methodology 

**Figure 2:**
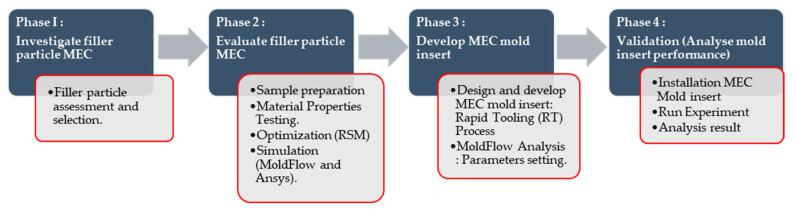
Flow chart.

The methodology of this research can be divided into four phases, as presented in Figure 2. The first phase starts with the investigation on the filler particles which emphasizes the literature review of previous studies on the use of filler particles as metal epoxy composite material. Based on previous studies, ALWA High Temperature resin M2200 manufactured by ALWA resin systems and porous slabs, Grunau, Germany for tooling applications and irregular-shaped brass and copper filler particles were selected for the purpose of improving the ability of mold inserts with regard to its durability and high thermal conductivity. In the second phase, the new MEC formulation was evaluated by producing specimens for various tests and characterization. The specimens were fabricated using filler particle materials of different metals mixed with epoxy resin. The physical characteristics of the newly formed MEC with different filler percentage compositions were evaluated mechanically and thermally for their suitability to be used as mold inserts in injection molding application. By using RSM, the optimum mixing parameters affecting the hardness of the MEC were determined. Experimental design to correlate mixing parameters with mechanical properties of MEC blend was based on two-level factorial designs generated using the optimization software (Design Expert 7, Stat-Ease Inc., Minneapolis, MN, USA). In the third phase, the mold inserts were designed using Computer-Aided Design (CAD) software (Solidworks 2014, Dassault Systems, S. A., Suresnes, France) with incorporating the cooling channels, gating systems, and considering the molded parts as tensile test specimens. Then, the design was simulated using Moldflow simulation software (Autodesk Moldflow Insight 2012, Moldflow, Melbourne, Australia) to obtain the recommended setting of processing parameters for the actual injection molding process. Next, the mold inserts were fabricated using a new MEC formulation and the performance of the mold inserts were evaluated experimentally using an injection molding machine (Nissei NEX1000, Nissei Plastic Industrial Co., LTD., Minamijo, Japan). Finally, in the last phase, the mold inserts were assembled as a sub-assembly and fitted into a standard mold base as a hybrid mold. The mold was tested and rectified before producing specimens for testing purposes. Tensile strength tests were performed on the specimen produced from the injection molding process. The molded part’s test was conducted to determine the effect of fillers on the molded parts.

### 2.1. Filler Particle Selection

Selection of an appropriate filler particle is very important in ensuring good performance of an MEC mold insert. Many studies have been conducted on various types of thermal and mechanical tests to examine the most influential parameters in the injection molding process, i.e., the cooling time [15,16,23,27,33]. In this research, the selection of brass and copper fillers are based on the properties of these materials which offer good thermal conductivity [23,33] while maintaining or increasing the compressive strength [16,27] compared to other types of fillers that have been used in previous studies [15,34]. In order to overcome these problems, many attempts have been made to load epoxy with irregular-shaped brass or copper fillers to evaluate their effectiveness in improving the properties of the epoxy. Table 1 presents the filler properties supplied by Chengdu Huarui Industrial Co., Ltd, Chendu, China, that were used in this study.

### 2.2. Sample Preparation

**Figure 3:**
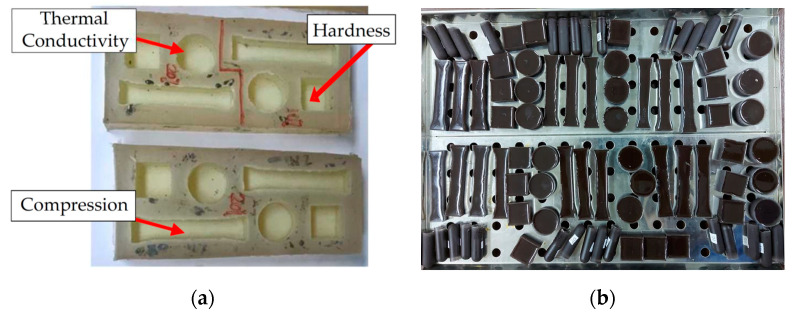
Specimen preparation: (**a**) silicone rubber mold; (**b**) MEC test samples.

Epoxy resin ALWA HT resin M2200 manufactured by ALWA resin systems and porous slabs, Gronau, Germany [35], were selected for the tooling matrix, mixed with metal fillers of brass (EB) and copper (EC). ALWA HT Resin is characterized by its good electronic insulation with outstanding properties such as high glass transition temperature, high distortion temperature, low thermal expansion and good chemical resistance to acids, alkalis and organic solvents. Table 2 tabulates the various compositions of the metal fillers (EB and EC), epoxy resin and hardener. Before mixing the epoxy resin with metal fillers, a silicon rubber mold was prepared with different geometries to produce the test samples for various testing parameters, such as hardness, compression strength, and thermal properties according to American Society for Testing and Materials (ASTM) standards [36,37,38], as shown in Figure 3. The mixture of epoxy resin with hardener material has the following specifications as provided by the manufacturer with a mixing ratio of 100:50 (Epoxy: Hardener) and 45 min to 1 h pot life in liquid form.

**Figure 4:**
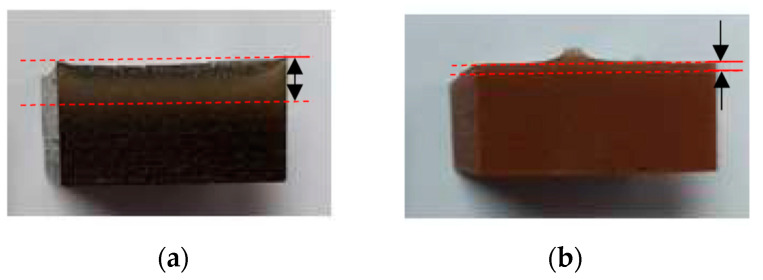
Filler sedimentation occurring at sample brass and copper: (**a**) brass filler; (**b**) copper filler.

After mixing the resin, hardener, and filler according to the prescribed composition, the mixture is stirred manually until the resulting mixture is well blended (within 5 to 10 min). Next, the mixture was de-gassed in the vacuum casting machine (CM2000, Cybron Technology (M) Sdn. Bhd, Peneng, Malaysia) and then poured into the silicone rubber mold. The mixture in the silicone mold was then pre-cured at room temperature for 24 h. Later, it was cured at 180 °C for 8 h in an oven (Memmert UM200, Memmert GmbH + Co. KG, Schwabach, Germany) following the manufacturer’s recommendation. It was found that after the sample is produced, sediment occurs where the mixed filler particles fall to the bottom, as shown in Figure 4. It can be seen that sedimentation occurs where brass filler showed more sedimentation compared to copper filler. Therefore, parameter optimization using RSM is required to obtain optimal sampling which can improve the material properties.

### 2.3. Material Properties Testing

The primary concern in selecting the MEC material is to match the material properties that have to be tested according to the ASTM standards (Table 3) so that the material requirement as a mold insert is met. These properties include physical, mechanical, and thermal with a combination of RT techniques. The tests were selected based on previous studies that focused on the mechanical and thermal properties of the MEC and its application in the injection molding process [15,39,40].

### 2.4. Response Surface Method

RSM is a statistical method to plan experiments, study the effect of process variables, obtain empirical input/output relationships, and determine optimal conditions [29,32]. RSM is one of the methods used for optimization which was introduced by Box and Wilson in 1951 [41]. It helps the researcher or experimenter to reach the goal of optimum response such as examining the hardness of samples in this research. Box–Behnken design (BBD) and central composite design (CCD) are RSM-based techniques to model the response in relation to the process parameters (control factors) for the manufacturing of quality composite specimens [30]. In this research, the selected factors and levels are tabulated in Table 4 which were developed using the optimization software.

Box–Behnken Design (BBD) was selected for RSM according to the number of variable parameters and levels, as shown in Table 4. It contains an embedded factorial design with 5 center points which allow for estimation of curvature. Therefore, 17 runs of experiments were generated.

### 2.5. Develop MEC Mold Insert

#### 2.5.1. Mold Insert Design

**Figure 5:**
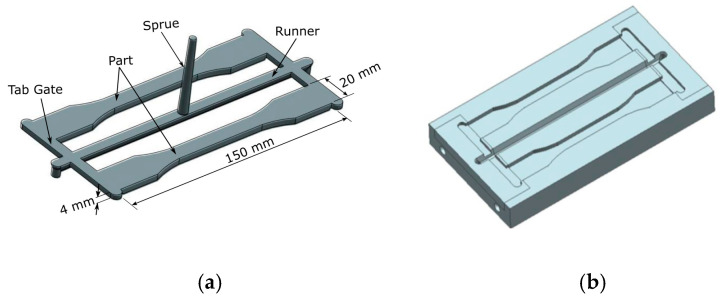
Mold inserts designed for the thick flat part: (**a**) part design; (**b**) core insert design.

The 3D model for the thick flat part was designed using Computer-Aided Design (CAD) based on the international standard for multi-purpose plastic injection test samples, ISO 3167: 2002 (E) [42]. The design phase of the mold emphasizes various important characteristics, such as the design of part shape, mold type, mold dimensions, material for mold inserts (core and cavity inserts), and the base of the mold which must be selected properly [6,13]. Figure 5 shows the mold insert design with two cavities that are used for this study. The result of fill + Pack analysis obtained from Moldflow simulation software was used to evaluate the packing pressure in the mold cavities and the recommended maximum packing pressure obtained to fill is 70 MPa according to the changes in the mass of the molded parts. Based on this value, the compression test of the MEC material should be higher than the required packaging pressure to avoid failure occurring on the MEC mold insert during the molding process.

#### 2.5.2. Fabrication of Mold Inserts for a Hybrid Mold

**Figure 6:**
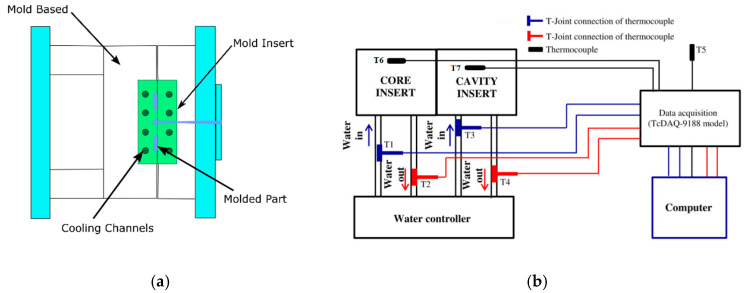
Hybrid mold design was instrumented with seven sensors: (**a**) hybrid mold design; (**b**) schematic diagram connection of the thermocouples.

The design of the hybrid mold and the cross-section of the assembly drawing are illustrated in Figure 6. The two-plate mold for the thick flat part as specimen was fabricated using sets of inserts with straight cooling channels. Two combinations of materials used were P20 as a mold base and MEC as mold inserts (core and cavity inserts). The fabrication steps for the MEC mold inserts include the degassing of the epoxy resin mixed with metal fillers in a vacuum chamber to remove air bubbles, the pre-curing at room temperature, and the post-curing in the oven based on the control factors of curing temperature and duration of curing time [12,13,27,43,44,45]. After the post-curing process, the finishing work using machining operations for fitting the mold standard components (ejector pins, sprue bushing, etc.) were performed to obtain the required dimensions and to allow adjustments for fitting the mold insert into the mold base. The hybrid mold was used with seven K-Type thermocouples to record the temperature profile of the ambient temperature (T5), the temperature of the coolants at the inlet (T1 and T3), and the outlet (T2 and T4) of the core and cavity, and the temperature of the core (T6) and the cavity (T7) during experimental work. The thermocouples are connected to the Data Acquisition System (DAQ) (TcDAQ-9188, National Instruments Corporation, Austin, TX, USA), and the recorded data are saved on the computer and then converted for further analysis into graphical form.

## 3. Results and Discussion

### 3.1. Thermal Conductivity Results

**Figure 7:**
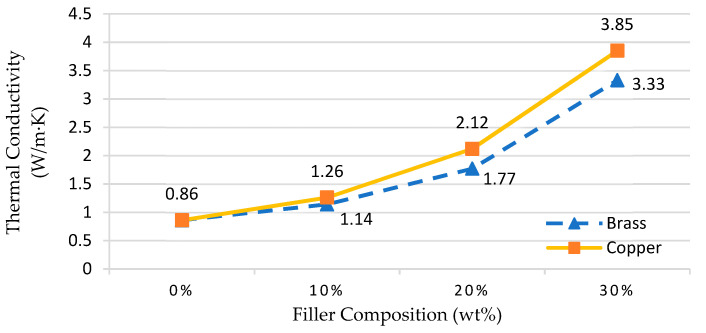
Effects of fillers on thermal conductivity.

The results indicate that the thermal conductivity of the copper filler is higher than the brass filler of irregular shape, as shown in Figure 7. The value of thermal conductivity for unfilled epoxy was in the range 0.6–0.9 W/m∙K, while commercially available aluminum filler epoxy composite is within 1.2 to 1.43 W/m∙K. The rapid increase in thermal conductivity can be attributed to the onset of interactions between irregular-shaped fillers when exceeding 10% by weight composition [33] compared to spherical-shaped fillers used by previous researchers [3,16,27,28,46]. A rapid increase in thermal conductivity was not observed at low compositions below 10 wt%, due to the diffusion effect in the bulk matrix almost without interaction. The most important finding is that irregular-shaped fillers can rapidly increase the thermal conductivity. Copper filler is a thermal conductivity enhancing element and this rapid increase in thermal conductivity is obviously due to the initial interaction of irregular particle filler shapes. This finding is similar to a previous study in which the thermal conductivity of the composites increased with the addition of the filler to the epoxy mixture [24].

### 3.2. Compression Results

**Figure 8:**
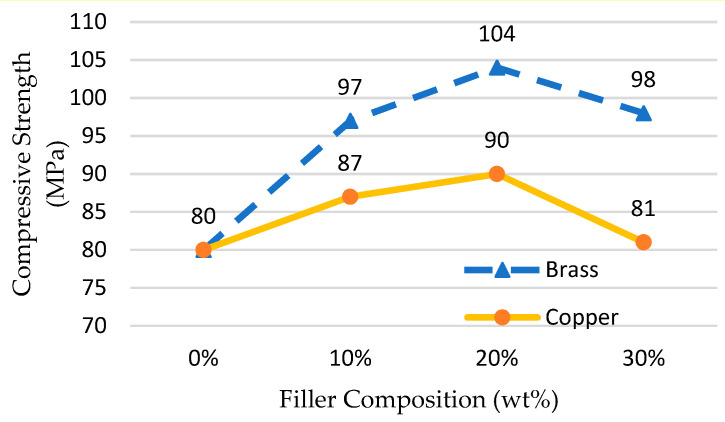
Effects of fillers on compressive strength.

In the injection molding process, the compression test strength of the MEC mold inserts is an important material property to withstand the clamping strength and packing pressure in the mold cavity and is useful for extending the life of the epoxy mold [47]. As presented in Figure 8, brass and copper fillers at 20 wt% composition indicate the highest average value of compressive strength of 104 and 90 MPa, respectively. Both fillers demonstrated a downward trend of its compressive strength after 20 wt% composition. However, the graph dropped gradually when adding filler composition more than 25–30% wt. Previous studies [15,23,27,48] on compression strength results indicated a non-linear trend between the filler weight percentage and the compression strength. Adding more fillers to the epoxy matrix beyond 20 wt% decreases the compressive strength because the epoxy matrix starts becoming more viscous, the porosity is increased and the fillers are agglomerated, consequently reducing the stiffness [15].

### 3.3. Hardness Results

**Figure 9:**
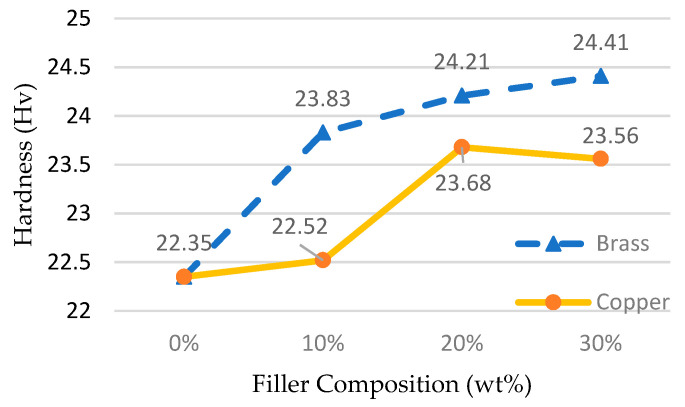
Effects of fillers on the hardness.

The hardness of the epoxy matrix composite is another important parameter affecting the durability and life of the mold. Figure 9 shows the results of hardness variations with different filler ratios. Brass fillers showed better hardness compared with copper fillers. The curve of hardness reflects an upward trend, having a positive slope. This result is similar to those of Senthilkumar et al. [28] and Srivastava and Verma [23], where hardness gradually increased with the increase in filler material.

### 3.4. Optimization Results

The result of the hardness test from the Box–Behnken Design (BBD) was generated using Design-Expert software to determine the influence of the mixing time, degassing time, and maximum curing temperature. The experimental results of the hardness were obtained from the 17 runs’ specimens. The values of hardness of the specimens were measured using Vickers Hardness and are tabulated in Table 5.

#### 3.4.1. Analysis of Results

ANOVA is a statistical analysis tool used to test the differences between two or more means across the different groups and to investigate the relationship between independent and dependent variables [30,32,41]. In this study, ANOVA is one of the initial methods in determining the factors that affect the hardness value of the specimens. In deciding the significance of the process parameters, *p*-value must be used along with the F statistic and F test to correctly interpret the results, of which the value of P must be smaller than the value of alpha (α = 0.05) [49]. The value of F can be determined based on the number of degrees of freedom and the total number of degrees of freedom of the factors with α = 0.05 [49]. Table 6 presents the results of the ANOVA obtained.

From the results of the analysis obtained, this model is significant due to the larger F-value compared to the *p*-value which is 0.0001. This model also shows non-significant lack of fit, which is good. On the other hand, from the F-value and Prob > F in the ANOVA result obtained as shown in Table 6, the most significant factor affecting the hardness is degassing time, which is 35.91%, followed by curing temperature which is 3.53% and mixing time which is 2.08%.

**Figure 10:**
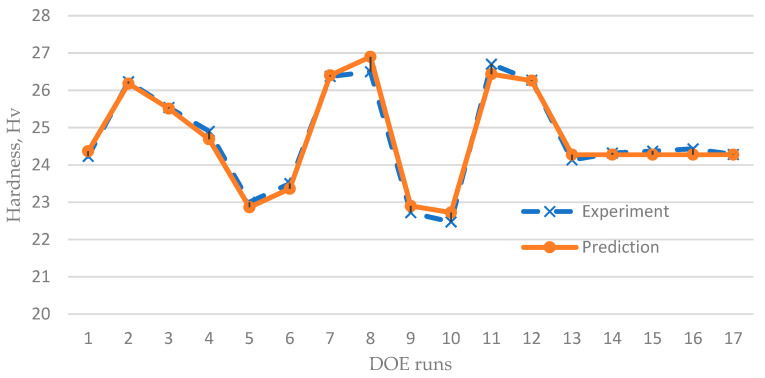
Experiment versus prediction results.

Based on the ANOVA results, high values of R^2^ and adjusted-R^2^ indicate a good explanation of the variability from the selected model (Table 7). It indicates that this model can predict the hardness result with 98.37% accuracy as shown in Figure 10.

#### 3.4.2. Confirmation Experiment.

Table 8 shows the optimal parameters that can maximize the hardness and the optimization result of RSM obtained by using Design Expert 7 software. Optimal hardness is achieved and proven by conducting validation experiments on the specimens produced according to the optimal parameters proposed.

## 4. Mold Inserts Trials

The fabrication of MEC mold inserts is based on the results of mechanical and thermal tests conducted by selecting the appropriate composition. The composition is set at 30 wt% due to the high thermal conductivity value offered (Figure 7) and the value of compression strength (Figure 8) is above the packing pressure required in the mold cavities which is more than 70 MPa (based on simulation studies using Moldflow simulation software). Based on the compression results, at 30 wt%, EB and EC mold inserts can withstand maximum pressures of 98 and 81 MPa, respectively. Several samples of mold inserts fabricated using MEC material are produced and tested using an injection molding machine. Acrylonitrile Butadiene Styrene (ABS) material was used as a plastic resin in these trials.

### 4.1. Simulation Results of Cooling Time for MEC Mold Inserts Using Simulation Software

**Figure 11:**
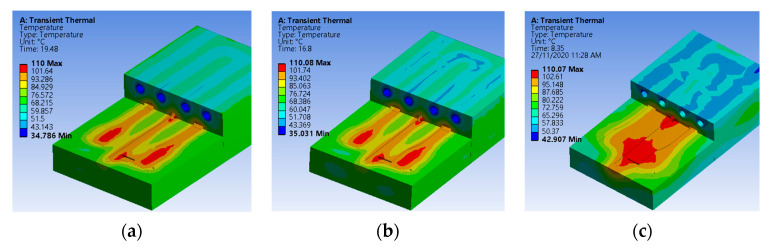
Result of transient thermal analysis: (**a**) EB mold insert; (**b**) EC mold insert; (**c**) P20 mold insert.

In this research, transient thermal analysis using simulation software (Ansys Fluent 180, ANSYS, Inc., Canonsburg, PA, USA) was used to evaluate the temperature distribution and cycle time during the molding cycle in the mold inserts. The setting of simulation parameters is divided into two steps; the heating phase to reach the melting temperature of 245 °C and a cooling phase to reach the ejection temperature of 110 °C before parts were ejected out from the mold. The convection coefficient for the cooling channel is 5098 W/m^2^ °C. Figure 11 and Table 9 show the results of temperature distribution and cycle time for EA, EB, and P20 mold inserts from Ansys Fluent simulation software. It can be seen that the EC mold insert offers a better cycle time than the EB mold insert. Table 9 shows that the difference in cycle time compared to the P20 mold insert is increasing two times and 2.33 times for EC and EB, respectively. Although the cycle time results obtained from MEC mold inserts are higher than P20 mold inserts, the use of this type of filler with irregular shape for low volume production has achieved less cycle time when compared to results from previous researchers [12,16,50] who used MEC mold inserts.

### 4.2. Experimental Results of Cooling Time for MEC Mold Inserts Using Machine Injection Molding

**Figure 12:**
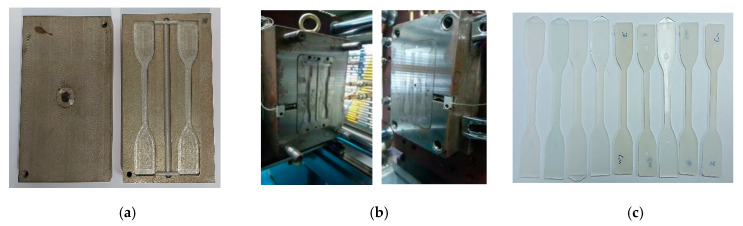
(**a**) MEC mold inserts; (**b**) mold inserts installed to mold based for injection molding process; (**c**) ejected parts.

The molded parts for the tensile strength are produced by different MEC mold insert materials including the P20 mold insert, and the injection process is used with a cooling system (Figure 12). The part was molded using Acrylonitrile Butadiene Styrene (ABS) material. From the actual trials, the total cycle time of the injection molding process using EB and EC mold inserts are 31 and 24 s, respectively. The dimensions and mass of the molded parts are consistent as well as the condition of the MEC mold without any defects after producing 100 shots. 

## 5. Conclusions

Mold inserts of injection mold for the injection molding process can be fabricated using alternative materials other than steel by using rapid tooling techniques for low volume production. MEC mold inserts can be used successfully to mold the plastic parts up to 100 shorts using ABS material without any defects on the mold inserts. By fabricating a hybrid mold in this research (MEC materials as mold inserts), most aspects related to the tool design, material performance, and influence on molded part properties are identified and understood.

The investigation on epoxy resin with the addition of metal fillers (brass and copper) was able to establish a new material as mold inserts for the injection molding process. The addition of brass (EB) and copper (EC) in epoxy resin is able to improve the material performance in terms of mechanical and thermal properties. Theoretically, the findings from this current research are:

1. The selection of the filler composition at 20–30 wt% as a mold insert is based on the maximum value of the compression strength test obtained.
2. The thermal conductivity and hardness of MEC increased with a positive slope when the composition of the filler on the epoxy matrix increased.
3. Brass fillers demonstrated a good effect for hardness and compression properties, while copper filler offered better thermal conductivity of the MEC produced.
4. The optimum parameters during the preparation of MEC material showed that the degassing time to remove bubbles from the mixture is the most important control factor (35.91%), which reduces voids and improves the structure of the epoxy matrix, followed by curing temperature (3.53%) and finally mixing time (2.08%).

The future work will continue with the multi-optimization of the compression and thermal conductivity properties by considering the combination of wt% composition for both brass and copper fillers. 

## Figures and Tables

**Table 1:** Filler properties.

Filler	Shape	Average Particle Size (mm)	Metal Contents (%)
Brass	Irregular	20–60 µm	95
Copper	Irregular	20–60 µm	99

**Table 2:** Composition of Metal Epoxy Composite (MEC) for 1 set specimen (thermal, compression and hardness).

No.	Mixing Composition (grams)							Total (100%)
	Metal Fillers			Epoxy Resin		Hardener		
1.	(EB or EC)	10%	7 g	60%	42 g	30%	21 g	70 g
2.		20%	14 g	53.3%	37.3 g	26.7%	18.7 g	70 g
3.		30%	21 g	46.7%	32.7 g	23.3%	16.3 g	70 g

**Table 3:** Mechanical and thermal properties test of epoxy with metal fillers.

Test	Standards	Equipment Used in this Study
Hardness	ASTM D2240-97 [38]	Vickers Hardness (Matsuzawa VMT-X Series, Matsuzawa Co., Ltd, Akita, Japan)
Compressive Strength	ASTM D695-96 [37]	Universal Testing Machine (Instron 5900 Series 50kN, Instron Corporation, Norwood, MA, USA)
Thermal conductivity	ASTM C1113 [36]	Thermal Properties Analyzer (Decagon KD2 Pro, Decagon Devices Inc., Pullman, WA, USA)

**Table 4:** Factors and levels of variable parameters.

Factor	Process Parameter	Unit	Low Level (−)	High Level (+)
A	Maximum Curing Temperature	°C	120	180
B	Mixing Time	Min	5	15
C	Degassing time	Min	10	40

**Table 5:** Experimental results of hardness strength.

Run	A) Curing Temp (°C)	B) Mixing Time, (min)	C) Degassing Time (min)	Hardness (Hv)
1	120	5	25	24.23
2	180	5	25	26.23
3	120	15	25	25.54
4	180	15	25	24.90
5	120	10	10	23.00
6	180	10	10	23.50
7	120	10	40	26.37
8	180	10	40	26.50
9	150	5	10	22.72
10	150	15	10	22.47
11	150	5	40	26.70
12	150	15	40	26.27
13	150	10	25	24.13
14	150	10	25	24.32
15	150	10	25	24.37
16	150	10	25	24.43
17	150	10	25	24.28

**Table 6:** Analysis of Variances (ANOVA) of Ra.

Source	Sum of Squares	df	Mean Square	F Value	*p*-Value Prob > F	
Model	29.37	6	4.89	100.61	<0.0001	significant
A-Curing Temperature	0.50	1	0.50	10.18	0.0097	
B-Mixing time	0.06	1	0.06	1.26	0.2880	
C-Degassing time	25.03	1	25.03	514.52	<0.0001	
AB	1.74	1	1.74	35.82	0.0001	
AA	1.55	1	1.55	31.96	0.0002	
BB	0.39	1	0.39	8.04	0.0177	
Residual	0.49	10	0.05			
Lack of Fit	0.44	6	0.07	5.65	0.0578	not significant
Pure Error	0.05	4	0.01			
Cor Total	29.85	16				

**Table 7:** Adequacy of the model.

Standard Deviations	R-Squared (%)	Adjusted R-Squared (%)
0.2205	0.9837	0.9739

**Table 8:** Optimal parameters for hardness strength.

Parameters	Units	Optimal Values
(A) Curing Temperature	°C	174.78
(B) Mixing time	Min	6.66
(C) Degassing time	Min	38.92
Response		
Hardness (Predicted)	Hv	27.09
Hardness (Experimental)	Hv	27.53

**Table 9:** Result of cooling time.

Mold Material	Cooling Time, tc (s)	Comparison of MEC with P20 Material
EB	19.48	EC > 2.33 time increasing of P20
EC	16.8	EC > 2 time increasing of P20
P20	8.35	-

## Data Availability

The data presented in this study are available on request from the corresponding author.
